# Portador Heterozigoto Composto de Hipercolesterolemia Familiar Causada por Variantes no LDLR

**DOI:** 10.36660/abc.20190582

**Published:** 2020-09-18

**Authors:** Heloisa Pamplona-Cunha, Marcela Freitas Medeiros, Thaís Cristine Marques Sincero, Isabela de Carlos Back, Edson Luiz da Silva

**Affiliations:** 1 Universidade Federal de Santa Catarina Florianópolis SC Brasil Universidade Federal de Santa Catarina,Florianópolis, SC - Brasil

**Keywords:** Hipercolesterolemia familiar, Hiperlipoproteinemia Tipo II, Triagem genética em cascata, Genotipagem, Aterosclerose, Heterozigoto Composto

## Abstract

A hipercolesterolemia familiar (HF) é uma doença genética causada por um defeito primário no gene que codifica o receptor da LDL. Mutações diferentes no mesmo gene caracterizam um heterozigoto composto, mas pouco se sabe sobre o fenótipo dos portadores. Portanto, neste estudo, descrevemos o rastreamento em cascata de uma família brasileira com essa característica. O caso-índice é um homem de 36 anos, com colesterol total (CT) de 360 mg/dL (9,3 mmol/L) e concentração de LDL-c de 259 mg/dL (6,7 mmol/L), além de xantomas de tendão de Aquiles, obesidade e pré-hipertensão. A genotipagem identificou as mutações 661G>A, 670G>A e 682G>A, no exon 4, e 919G>A, no exon 6. A mesma mutação no exon 4 foi observada no filho do caso-índice (7 anos), que também tem hipercolesterolemia e xantomas tendinosos, ao passo que a filha do caso-índice (9 anos) apresenta mutação no exon 6 e hiperlipidemia, sem xantomas. Em suma, este relato permite uma melhor compreensão acerca da base molecular da HF no Brasil, um país multirracial, onde é esperada uma população heterogênea.

## Introdução

O aumento dos níveis plasmáticos de colesterol total (CT) e da lipoproteína-colesterol de baixa densidade (LDL-c) ocorre em pacientes com formas graves e precoces de hipercolesterolemia familiar (HF), uma doença genética geralmente resultante de mutações no gene LDLR, que codifica o receptor LDL.^1^ A detecção da mutação patogênica é o padrão ouro para o diagnóstico de HF, e a forma mais eficiente de triagem é o rastreamento dos parentes de um paciente já diagnosticado (caso-índice).^1^

A presença de mutações distintas no mesmo gene caracteriza o indivíduo como heterozigoto composto.^2^^-^^4^ Embora as consequências clínicas das mutações heterozigotas e homozigotas sejam descritas frequentemente, pouco se sabe sobre os fenótipos de portadores heterozigotos compostos.^2^^-^^4^ A grande sobreposição de fenótipos dentro do espectro de portadores de mutação relacionadas à HF pode explicar a subnotificação dos heterozigotos compostos e sugere que este diagnóstico é facilmente perdido.^2^

Levando-se em consideração que encontramos apenas um relato de heterozigoto composto para HF no Brasil,^5^ descrevemos neste estudo o rastreamento de uma família brasileira com essa característica. Os métodos aplicados estão descritos no Arquivo Suplementar 1.

## Resultados

Todos os participantes assinaram um termo de consentimento livre e esclarecido do protocolo de pesquisa, incluindo os testes genéticos, e o Comitê de Ética em Pesquisa com Seres Humanos da Universidade Federal de Santa Catarina aprovou o estudo (CAAE: 54585416.1.0000.0121).

O Arquivo Suplementar 2 mostra a árvore genealógica do caso-índice (indivíduo I) e seus familiares. O indivíduo I é um homem de 36 anos de idade. Apesar de fazer uso diário de 20 mg de Sinvastatina, o caso-índice apresentou CT de 360 mg/dL (9,3 mmol/L), um valor de LDL-C de 260 mg/dL (6,7 mmol/L), correspondente a 80,4% da fração pequena e densa da LDL (sd-LDL), medida após a precipitação das demais lipoproteínas apoB^6^ (Arquivo Suplementar 1). Também apresentou concentrações plasmáticas elevadas de não-HDL-c, triglicérides e ApoB, além de níveis baixos de HDL-c. O paciente é fumante, sedentário (Arquivo Suplementar 3), obeso com obesidade abdominal e pré-hipertenso. O ultrassom Doppler carotídeo não mostrou aumento da espessura íntima-média, o que não exclui aterosclerose subclínica. Infelizmente, neste estudo, a aterosclerose coronária subclínica não foi avaliada através de angiografia por tomografia computadorizada (TC). Foram identificados xantomas de tendão de Aquiles, e o diagnóstico clínico de HF foi definitivo.

O caso-índice e sua esposa (indivíduo II) relataram histórico familiar de parente de primeiro grau com doença da artéria coronariana (DAC) precoce e concentrações elevadas de CT, mas nenhum caso de doença cardiovascular (DCV) precoce.

O genótipo LDLR do caso-índice revelou três mutações no exon 4 (661G>A, 670G>A, 682G>A) e uma no exon 6 (919G>A).

O indivíduo II não apresentava sinais ou sintomas de HF e não tinha diagnóstico clínico. O rastreamento genético em cascada permitiu a identificação da mutação 919G>A na filha do caso-índice (indivíduo III; 9 anos), e as mutações 661G>A, 670G>A, e 682G>A no filho (indivíduo IV; 7 anos), tendo sido confirmado, assim, o diagnóstico de HF. Ambos os filhos apresentaram hipercolesterolemia (CT 394 and 332 mg/dL (10,2 e 8,6 mmol/L); LDL-c 329 e 286 mg/dL (8,5 e 7,4 mmol/L), e sd-LDL-c 63,5 e 90,5% para os indivíduos III e IV, respectivamente), com índices elevados de ApoB, indicando um padrão mais aterogênico. Além disso, o indivíduo IV apresentava xantomas na mão direita (interdigitais) e no cotovelo esquerdo (Arquivo Suplementar 3). Nenhum dos filhos estava sob tratamento hipolipemiante, e não havia sinais de DAC clínica. O ultrassom com Doppler não revelou nenhuma obstrução na artéria carótida nos indivíduos dessa família. Embora seja provável que eles não tivessem DAC, não é possível afirmar com 100% de certeza devido à falta da angiografia por TC.

Neste estudo, o diagnóstico de FH foi realizado com base nos critérios da Dutch Lipid Clinic Network (DLCN). Deve-se salientar que esse critério é útil apenas para adultos e o diagnóstico nas crianças pode ser subestimado. Levando-se em consideração o critério, mas não o teste genético, os indivíduos I e IV obtiveram 14 pontos e o diagnóstico foi de HF clínica. Por outro lado, o sujeito III obteve apenas oito pontos (diagnóstico provável). Após a identificação das variantes através do teste genético, todos os três sujeitos tiveram mais que oito pontos e foram considerados portadores de HF.

## Discussão

O caso-índice apresentou quatro mutações no gene LDLR, o que caracteriza um heterozigoto composto. O caso-índice provavelmente apresentou um fenótipo leve de HF devido à terapia hipolipemiante. Além disso, apresentava comorbidades, tais como obesidade e hipertensão, o que pode complicar o prognóstico. A aterosclerose coronária subclínica não foi avaliada neste estudo. O rastreamento em cascata permitiu a identificação de dislipidemia e o diagnóstico da HF nos filhos do caso-índice. Curiosamente, todos os indivíduos estudados apresentaram níveis elevados de sd-LDL, ao contrário de relatos anteriores, que mostraram uma prevalência de partículas grandes e flutuantes de LDL em pacientes com HF.^7^ Estudos adicionais são necessários para esclarecer este achado.

A mutação 661G>A substitui o códon GAC por AAC, modificando o aminoácido aspartato por asparagina, na posição 221 da cadeia proteica. Consequentemente, ocorre a substituição de um aminoácido carregado negativamente por um aminoácido fracamente bipolar, o que afeta a atividade de ligação proteína-ligante.^8^

A mutação 670G>A corresponde à troca do códon GAC pelo AAC na posição 224 da cadeia proteica, levando à substituição do aminoácido aspartato pela asparagina. A mutação gera um receptor r com menos de 2% de sua atividade normal.^9^

No caso da mutação heterozigota 682G>A, o códon GAG é substituído pelo AAG, levando à substituição do ácido glutâmico por lisina na posição 228. Essa mutação origina a troca de um aminoácido básico por um aminoácido ácido, o que prejudica o transporte do receptor da LDL do retículo endoplasmático para a superfície celular.^10^ resultando em um receptor LDL com menos de 2% de sua atividade normal.^11^ As mutações na extremidade 3’ do exon 4 do gene LDLR são uma causa muito comum de HF, e alterações de base única idênticas nessa região curta ocorrem em diferentes populações, especialmente em dinucleotídeos CG. Assim, é pouco provável que a mutação tenha sido herdada de um ancestral comum.^11^

A mutação 919G>A modifica o códon GAT, correspondente ao aspartato, para AAT, correspondente à asparagina, na posição 307 da cadeia proteica.^12^ A análise *in silico* indicou que essa mutação é provavelmente patogênica. O exon 4 não foi genotipado na filha do caso-índice e ela pode ter mutações semelhantes às do pai. Apesar dos níveis similares de lipídios séricos, ao contrário do pai e do irmão, a filha (indivíduo IV) não apresentou xantomas.

As mutações 661G>A, 670G>A e 682G>A foram classificadas como patogênicas/provavelmente patogênicas de acordo com o ClinVar. Por outro lado, a mutação 919G>A possui interpretações conflitantes de patogenicidade (provavelmente patogênico/significado incerto). No entanto, deve-se observar que achados *in silico* não provam a patogenicidade.^13^

Há relatos de que mutações homozigotas ou heterozigotas compostas no LDLR, além de mutações duplas no LDLR, estão associadas a níveis mais elevados de LDL-c, xantomatose mais extensa e DAC prematura mais grave em relação às mutações heterozigotas simples.^3^^,^^4^ Entretanto, todos os indivíduos que participaram deste estudo apresentaram fenótipos leves. Contudo, o caso-índice e seus filhos não foram submetidos à avaliação para a presença de aterosclerose coronária subclínica. Todos os indivíduos foram encaminhados a um cardiologista para receberem tratamento e acompanhamento apropriados.

Não podemos afirmar se um diagnóstico genético “duplo” teria relevância clínica quando os pacientes já foram diagnosticados e estão sendo tratados adequadamente com terapia hipolipemiante.^2^ No entanto, é de importância clínica perceber que essa população deve ser informada sobre a importância da sua herança genética, uma vez que a herança de distúrbios monogênicos combinados é mais grave e está associada a um maior risco de desenvolvimento de DCV, requerendo, assim, maior cuidado.^2^

Os achados relatados neste estudo ajudam a elucidar as bases moleculares da HF no Brasil, uma vez que há apenas nove estudos disponíveis sobre o rastreamento molecular da HF^5^ e, levando-se em consideração que este é um país multirracial, é esperada uma população heterogênea. O objetivo desta pesquisa é contribuir para o diagnóstico genético e o aconselhamento de pacientes com HF.
