# Supplementary material for: Portador Heterozigoto Composto de Hipercolesterolemia Familiar Causada por Variantes no LDLR
Source: Arq Bras Cardiol. 2020 Sep 18;115(3):587–9. [Article in Portuguese] doi: 10.36660/abc.20190582 (PMC9363081; doi:10.36660/abc.20190582)
Supplement: Translation

# Translation: Compound Heterozygous Familial Hypercholesterolemia Caused by LDLR Variants

**Authors:** Heloisa Pamplona-Cunha, Marcela Freitas Medeiros, Thaís Cristine Marques Sincero, Isabela de Carlos Back, Edson Luiz da Silva

**Affiliations:** 1 Brazil Universidade Federal de Santa Catarina, Florianópolis, SC - Brazil

**Keywords:** Familial hypercholesterolemia, Hyperlipoproteinemia Type II, Genetic Cascade Screening, Genotyping, Atherosclerosis, Compound Heterozygous

## Abstract

Familial hypercholesterolemia (FH) is a genetic disease caused by a primary defect in the LDL-receptor gene. Distinct variants in the same gene characterize a compound heterozygote, but little is known about the phenotypes of the carriers. Therefore, herein, we describe the cascade screening of a Brazilian family with this characteristic. The index case, a 36-year-old male, had a total cholesterol level of 360 mg/dL (9.3 mmol/L) and LDL-c value of 259 mg/dL (6.7 mmol/L), in addition to Achilles tendon xanthomas, obesity and prehypertension. Genotyping identified the variants 661G>A, 670G>A, 682G>A in exon 4 and 919G>A in exon 6. The same variant in exon 4 was found in the index case’s son (7-y), who also had hypercholesterolemia and xanthomas, while the index case’s daughter (9-y) had the variant in exon 6 and hyperlipidemia, without xanthomas. In summary, this report allows for a better insight into the molecular basis of FH in Brazil, a multi-racial country where a heterogeneous population is expected.

## Introduction

Increased plasma levels of total cholesterol (TC) and low-density lipoprotein-cholesterol (LDL-c) occur in patients with severe and early forms of familial hypercholesterolemia (FH), a genetic disease usually due to alterations in the *LDLR* gene, which encodes the LDL receptor.^1^ Detection of a pathogenic variant is the gold standard for FH diagnosis and the most efficient form of diagnosis is the screening of relatives of a diagnosed patient (index case).^1^

The presence of distinct variants in the same gene characterizes the individual as a compound heterozygote.^2^^-^^4^ Although the clinical consequences of heterozygous and homozygous variants are often described, little is known about the phenotypes of compound heterozygote carriers.^2^^-^^4^ The large overlap of phenotypes within the spectrum of FH-related variant carriers may explain the underreporting of compound heterozygosity and suggests that this diagnosis is easily missed.^2^

Considering that we found only one report of compound heterozygosity for FH in Brazil,^5^ we describe herein the screening of a Brazilian family with this characteristic. The methods applied are available in Supplemental File 1.

## Results

All participants gave written informed consent for the study protocol, including the genetic tests, and the Human Studies Committee of the Federal University of Santa Catarina approved the study (CAAE: 54585416.1.0000.0121).

Supplemental File 2 shows the pedigree of the index case (subject I) and his family. Subject I is a 36-year-old male. Despite a daily intake of 20 mg Simvastatin, the index case presented a TC of 360 mg/dL (9.3 mmol/L) and LDL-c value of 260 mg/dL (6.7 mmol/L), corresponding to 80.4% small dense-LDL-cholesterol (sd-LDL-c), measured after precipitation of the other apoB-lipoproteins^6^ (Supplemental File 1). He also had high concentrations of non-HDL-c, triglycerides and ApoB, in addition to low HDL-c levels. The patient is a smoker, sedentary (Supplemental File 3), obese, and has abdominal obesity and prehypertension. The carotid Doppler ultrasound did not reveal increased intima-media thickness, which does not exclude subclinical atherosclerosis. Unfortunately, in this study, subclinical coronary atherosclerosis was not evaluated by computed tomography (CT) angiography. Xanthomas were identified in the Achilles tendon and the clinical diagnosis of FH was definitive.

The index case and his wife (subject II) reported having a family history of a first-degree relative with early coronary artery disease (CAD) and high TC concentrations, but no cases of early cardiovascular disease (CVD).

The genotype of the index case *LDLR* revealed three variants in the exon 4 (661G>A, 670G>A, 682G>A) and one in the exon 6 (919G>A).

Subject II had no signs or symptoms of FH and had no clinical diagnosis. Genetic cascade screening allowed for the identification of the variant 919G>A in the index case’s daughter (subject III; 9-y), and the variants 661G>A, 670G>A, and 682G>A in his son (subject IV; 7-y), thereby confirming the diagnosis of FH. Both children presented hypercholesterolemia (394 and 332 mg/dL (10.2 and 8.6 mmol/L) TC; 329 and 286 mg/dL (8.5 and 7.4 mmol/L) LDL-c, and 63.5 and 90.5% sd-LDL-c for subjects III and IV, respectively), with high levels of ApoB, indicating a more atherogenic pattern. Furthermore, subject IV presented xanthomas in the right hand (interdigital) and left elbow (Supplemental File 3). Both children were not on lipid-lowering therapy and had no signs of clinical CAD. Doppler ultrasound did not reveal any carotid artery obstruction in the subjects of this family. Although it is probable that they do not have CAD, it is not possible to be one hundred percent sure in the absence of CT angiography.

In this study, the FH diagnosis was performed as defined by the Dutch Lipid Clinical Network (DLCN). It should be noted that this criterion is useful for adults only and the diagnosis in children may be underestimated. Considering the criterion, but not the genetic test, subjects I and IV obtained 14 points and the diagnosis was clinical FH. On the other hand, subject III obtained only eight points (probable diagnosis). After the identification of variants by genetic testing, all three subjects had more than eight points and were considered FH carriers.

## Discussion

The index case showed four variants in the *LDLR*, which characterizes a compound heterozygote. The index case probably showed a mild FH phenotype due to the lipid-lowering therapy. Moreover, he presented comorbidities, such as obesity and hypertension, which may complicate his prognosis. Subclinical coronary atherosclerosis was not evaluated in this study. The cascade screening allowed for the identification of dyslipidemia and the diagnosis of FH in the children of the index case. Surprisingly, all subjects studied had high levels of sd-LDL, in contrast to previous reports that showed a prevalence of large buoyant LDL particles in FH patients.^7^ Further studies are needed to clarify this finding.

The variant 661G>A replaces the codon GAC with AAC, changing the amino acid aspartate to asparagine at position 221 of the protein chain. Therefore, the substitution of a negatively charged amino acid by a weakly bipolar amino acid occurs, which affects the protein-ligand binding activity.^8^

The variant 670G>A corresponds to the substitution of codon GAC with AAC at position 224 of the protein chain, leading to the replacement of aspartate with asparagine. The variant generates a receptor with less than 2% of its normal activity.^9^

In the case of the heterozygous variant 682G>A, the codon GAG is replaced with AAG, leading to the substitution of glutamic acid with lysine at position 228. This variant causes the exchange of a basic amino acid for an acidic amino acid, which impairs the transport of the receptor from the endoplasmic reticulum to the cell surface,^10^ resulting in an LDL-R with less than 2% normal activity.^11^ Variants in the 3’ end of exon 4 of the LDLR gene are a very common cause of FH, and identical single base changes in this short region, especially in the CG dinucleotides, occur in different populations. Thus, it is highly unlikely that the variant was inherited from a common ancestor.^11^

The variant 919G>A modifies the codon GAT, corresponding to aspartate, to AAT, corresponding to asparagine, at position 307 of the protein chain.^12^*In silico* analysis indicated that this variant is probably pathogenic. Exon 4 was not genotyped in the daughter of the index case and she may have similar variants as her father. In spite of the comparable levels of serum lipids, in contrast to her father and brother, the daughter (subject IV) did not present xanthomas.

The variants 661G>A, 670G>A and 682G>A have been classified as pathogenic/likely pathogenic according to ClinVar. On the other hand, the variant 919G>A has a conflicting interpretation of pathogenicity (likely pathogenic/uncertain significance). However, it should be noted that *in silico* findings do not prove pathogenicity.^13^

It has been reported that compound homozygous or heterozygous *LDLR* variants, in addition to double heterozygous *LDLR* variants, are associated with higher levels of LDL-c, more extensive xanthomatosis, and more severe premature CAD than simple heterozygotes.^3^^,^^4^ However, all subjects that participated in this study presented a mild phenotype. Nevertheless, the index case and his children have not been evaluated for subclinical coronary atherosclerosis. All subjects were referred to a cardiologist to receive appropriate treatment and follow-up.

The question remains whether a “double” genetic diagnosis has clinical relevance when patients have already been diagnosed and are being adequately treated with lipid-lowering therapy.^2^ It is, however, of clinical importance to realize that this population must be advised of the importance regarding their genetic inheritance, since the combined monogenic disorder inheritance is more severe and is associated with a greater risk of developing CVD, thus requiring greater care.^2^

The findings reported herein provide an aid to gaining a better insight into the molecular basis of FH in Brazil, since there are only nine studies available on FH^5^ molecular screening and, considering that this is a multi-racial country, a heterogeneous population is expected. The aim of this research is to contribute to the genetic diagnosis and counseling of FH patients.
